# Discrimination and Content Analysis of Fritillaria Using Near Infrared Spectroscopy

**DOI:** 10.1155/2015/752162

**Published:** 2015-02-18

**Authors:** Yu Meng, Shisheng Wang, Rui Cai, Bohai Jiang, Weijie Zhao

**Affiliations:** ^1^State Key Laboratory of Fine Chemicals, Dalian University of Technology, Dalian 116024, China; ^2^School of Pharmaceutical Science and Technology, Dalian University of Technology, Dalian 116024, China

## Abstract

Fritillaria is a traditional Chinese herbal medicine which can be used to moisten the lungs. The objective of this study is to develop simple, accurate, and solvent-free methods to discriminate and quantify Fritillaria herbs from seven different origins. Near infrared spectroscopy (NIRS) methods are established for the rapid discrimination of seven different Fritillaria samples and quantitative analysis of their total alkaloids. The scaling to first range method and the partial least square (PLS) method are used for the establishment of qualitative and quantitative analysis models. As a result of evaluation for the qualitative NIR model, the selectivity values between groups are always above 2, and the mistaken judgment rate of fifteen samples in prediction sets was zero. This means that the NIR model can be used to distinguish different species of Fritillaria herbs. The established quantitative NIR model can accurately predict the content of total alkaloids from Fritillaria samples.

## 1. Introduction

**Figure 1:**
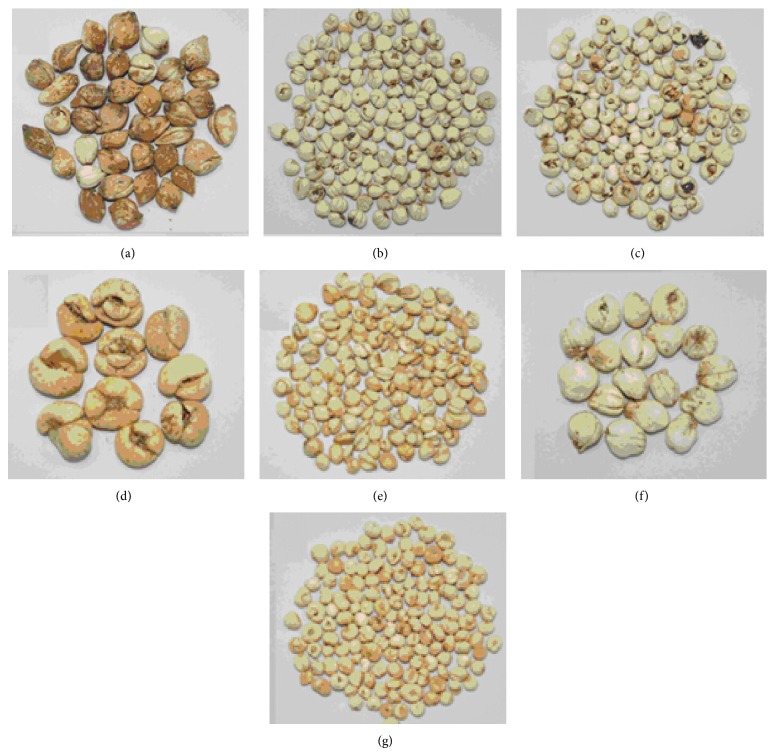
Seven kinds of Fritillaria materials used in China. (a)–(g) were* Fritillaria delavayi *Franch,* F. unibraacteate *Hsiao et. K. C. Hsia,* F. cirrhosa *D. Don,* F. thunbergii *Miq. (big),* F. thunbergii *Miq. (small),* F. pallidiflora *Schrenk, and* F. ussuriensis *Maxim, respectively.

The plant Fritillaria belongs to the genus* Fritillaria* of the Liliaceae family (known in English as fritillary). This family is native to the temperate regions of the Northern Hemisphere [1]. The Fritillaria bulb has many medical uses, especially in traditional Chinese medicine, such as clearing heat, moistening lung, eliminating phlegm, and relieving cough, and for the treatment of mammary abscess, pulmonary dryness, and chronic cough [2]. The genus* Fritillaria* includes about 130 species worldwide. The different species each have unique effects. Bulbus Fritillariae Cirrhosae and* F. thunbergii* Miq. are widely used in China for different purposes.* F. thunbergii* Miq. is good for detoxification, eliminating phlegm, releasing stagnated lung-qi, and treating “wind-fire syndrome” in traditional Chinese medicine. Bulbus Fritillariae Cirrhosae has a sweet flavor and is used for lung tonic and treatment of cough. In northeast and northwest of China, Bulbus Fritillariae Cirrhosae is always replaced by* F. pallidiflora* and* F. ussuriensis* as a medication. Because of its various subspecies and diverse effects, Fritillaria is easily confused and misused (shown in Figure 1). Most of the species are similar in morphology, making it hard to distinguish one from another. Since the existing identification methods are inconvenient and troublesome, developing a fast, simple, accurate, and solvent-free method to discriminate the varieties of Fritillaria is very useful.

**Figure 2:**
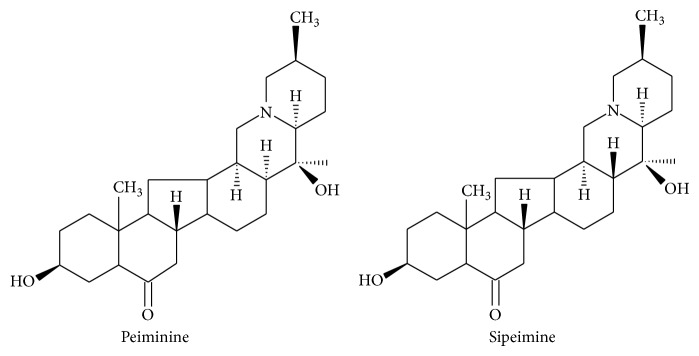
Structures of peiminine and sipeimine.

Steroidal alkaloids are the main bioactive constituents in genus* Fritillaria*; the content of total alkaloids is the major index of quality evaluation of Fritillaria materials. The type and content of alkaloids differ among species. BulbusFritillariae Thunbergii mainly contains peimine, peiminine, sipeimine, peimisine, and peimiphine [3]; Bulbus Fritillariae Cirrhosae contains fritimine, minpeimine, peiminine, sonpeimine, chinpeimine, and sipeimine [4]; Bulbus Fritillariae Pallidiflorae contains fritimine, sipeimine, peimisine, and oxynitrides of sipeimine [5]. Among the steroidal alkaloids in fritillaries, peiminine and sipeimine exist in every species of Fritillaria and can thus be selected as a standard to determine the total alkaloid content (Figure 2). The main measurement methods include the ultraviolet spectrum [4, 5], ELSD [6], and colorimetry [7].

Near infrared (NIR) light refers to the electromagnetic waves located between visible light and intermediate infrared light. Its wavelength is between 780 and 2500 nm (12000 to 4000 cm^−1^). The NIR spectrum reflects the absorption of frequency multiplication and basic frequency of chemical groups, such as C-N, N-H, and O-H [8, 9]. Thanks to the rapid development of chemometrics over the past ten years, researchers can now analyze multivariate data. With the development of these techniques, the application area of the NIR spectrum has been expanded, and lots of information in NIRS can be explained, while a multicomponent sample is analyzed. Today, NIRS methods are widely used in quantitative and qualitative analysis of pharmaceutical raw material, package material, and pharmaceutical products [10–13]. Hu et al. established a near infrared diffuse reflectance spectroscopy with PLS method to quantitatively determine the* Fritillaria thunbergii* Miq. mixed into* Fritillaria cirrhosa* D. Don [14]. Gao et al. identified three fake Fritillaria from seven certified Fritillaria by using near infrared diffuse reflectance spectroscopy, but the seven certified Fritillaria could not be clearly distinguished [15]. Herein, we established a fast, accurate, and simple NIR method to discriminate seven species of Fritillaria and their total alkaloid content.

## 2. Experimental

### 2.1. Materials and Instruments

#### 2.1.1. Herbal Materials

*Fritillaria delavayi *Franch (Sichuan Province)*, F. unibraacteate *Hsiao et. K. C. Hsia (Sichuan Province)*, F. cirrhosa *D. Don (Sichuan Province)*, F. thunbergii *Miq. (big) (Zhejiang Province)*, F. thunbergii *Miq. (small) (Zhejiang Province)*, F. pallidiflora *Schrenk (Xinjiang Province), and* F. ussuriensis *Maxim (Heilongjiang Province) were collected from the place of origin in 2007 and authenticated by Daixian Chen who is a chief pharmacist of traditional Chinese medicine from Dalian Institute for Food and Drug Control.

#### 2.1.2. Instruments

The Bruker NIR Spectrometer (MPA; Bruker Optics, Germany), equipped with an indium gallium arsenide (InGaAs) and a lead sulfide (PbS) detector, was used in the experiments; Unicam Helios Alpha 9423 UV Vis Double Beam Spectrophotometer (UNICAM) and Mettler Toledo AG245 Dual Range Analytical Scale Balance (Mettler) were also used.

#### 2.1.3. Reagent

Methanol (HPLC grade, Merck), ultrapure water (Millipore), chloroform (Merck) and potassium biphthalate (Merck) were used. The standard substances, peiminine (20 mg, batch number 110751-200607) and sipeimine (20 mg, batch number 110767-200504), were both purchased from National Institutes for Food and Drug Control.

### 2.2. Sample Pretreatment

The seven Fritillaria samples were crushed separately and then ground into fine powder. Each powder was sieved on a 100 mesh and dried under 60°C for 12 h to yield a 6 g sample. The seven samples were reserved in a dry atmosphere.

### 2.3. Content Determination of the Total Alkaloids by UV Spectroscopy

#### 2.3.1. Determination of Standard Curve

About 2.0 mg of standard peiminine was dissolved in 100 mL chloroform, and the standard peiminine solutions of 1.0, 2.0, 3.0, 4.0, and 5.0 mL were set in 60 mL separating funnels, respectively. Chloroform was then added to 8.0 mL, before 2.0 mL of pH 5 buffer solution and 2.0 mL of 0.001 mol/L bromothymol blue were added. After shaking and standing, the chloroform layer was filtered, and the aqueous part was washed with chloroform. The combined chloroform solution was diluted to 10 mL, and the absorbance was measured at 410 nm with blank control as a reference.

The linear regression equation was obtained as follows:

(1) $$\begin{array}{c} A=0.000541+0.040201C\;\left(r=0.9998\right). \end{array}$$

#### 2.3.2. Preparation of Total Alkaloids from Fritillaries

Seven samples of different fritillary powders, each weighing about 2 g, were wetted with 18% ammonia water 2 mL for 1 h in the conical flask with a plug and then extracted twice with a mixed solvent (diethyl ether/chloroform/95% ethanol 25 : 8 : 2.5) in ultrasonic cleaner (30 and 15 mL, resp., for 30 min each time). After washing residues with 15 mL of mixed solvent, the combined solution was evaporated to dryness at 60°C and then dissolved with chloroform to 10 mL for analysis.

#### 2.3.3. Content Determination of Total Alkaloids from Fritillaries

Each sample of total alkaloid solution 0.8 mL was evaporated to dryness, and chloroform 8.0 mL, a pH 5 buffer solution of 2.0 mL, 0.001 mol/L of bromothymol blue 2.0 mL were added. After a severe shake and stationary placement, the chloroform layer was filtered, and the aqueous layer was washed by chloroform. The combined chloroform solution was diluted to 10 mL. The absorbance of the sample at 410 nm was measured immediately.

UV absorption regression equation of peiminine was obtained as follows:

(2) $$\begin{array}{c} A=0.000541+0.040201C\;\left(r=0.9998\right). \end{array}$$

The total alkaloid content in Fritillaria can be calculated by formula (3), where *A* is the UV absorbance at 410 nm, *N* is the dilution times 125, and *W* is the sample weight (g):

(3) $$\begin{array}{c} \text{Total}\;\text{alkaloids}\left(\%\right)=\frac{\left(A-0.000541\right)}{W\times 106\times 0.040201}\times N. \end{array}$$

### 2.4. NIR Spectrum Collection

Consider the following.

**Figure 3:**
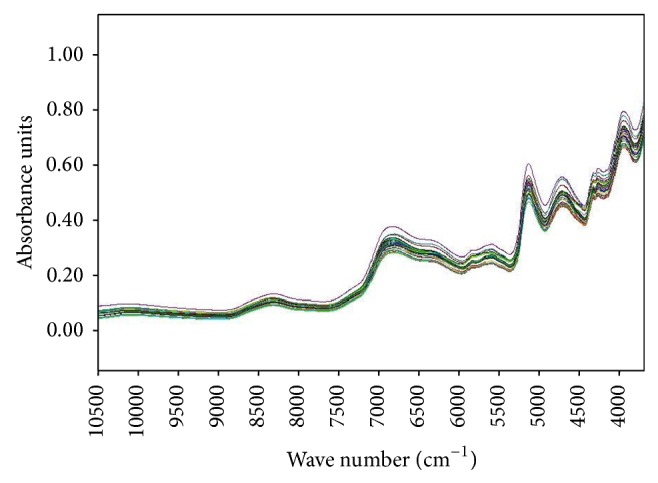
The original NIR spectrum of seven Fritillaria samples.

 Instrument condition: spectral resolution, 4 cm^−1^. Sample scanning times: 32. Background scanning times: 32. Scanning range: 12000~3700 cm^−1^.
 Procedure: put some powder in the measurement glass. The original spectrum of each sample is collected by using diffuse reflection method for 3 times and the average spectrum is obtained. The NIR spectra of seven Fritillaria samples are as follows (Figure 3).

### 2.5. Data Analysis

The calibration models of quantitative and qualitative analysis were developed by OPUS 6.5 software from Bruker Optics and MATLAB 7.1 software. For quantitative analysis, a PLS regression and a leave-one-out (LOO) cross validation were used to construct calibration models. The performance of the calibration models was evaluated by comparing the NIR predicted values with the UV measured values and evaluating parameters including the root means square error of cross validation (RMSECV), root mean square error of prediction (RMSEP), and correlation coefficient (*R* ^2^). For the validation set, square error of prediction (SEP) was used to verify the precision of the developed NIRS model. Detailed computing formula of above mentioned parameters can be found in [16].

The identification model was established by utilizing algorithms such as factorization and scaling to first range, which were provided by OPUS 6.5. Factor analysis was carried out on the spectra, and the spectrum (*a*) can be expressed as a linear combination of orthogonal factor spectra (*f*_*i*_) and *T*_*i*_: *a* = *T*_1*a*_ *f*_1_ + *T*_2*a*_ *f*_2_ + *T*_3*a*_ *f*_3_ + ⋯+*T*_*ia*_ *f*_*i*_. Then the spectrum distance (*D*) between the unknown spectra and the average spectrum in the calibration set (Hit) was calculated according to formula (4), and the threshold for each category (*D*_*T*_) was obtained according to formula (5), where *S*_Dev_ is the standard deviation of Hit and *X* is a coefficient with 0.25 being selected by experience. When *D* is less than *D*_*T*_, the matching degree between the spectra of the unknown sample and calibration samples is high, which suggests the sample spectrum and reference spectra can be considered one category [13]:

(4) $$\begin{array}{c} D=\sqrt{\sum_{i}{\left[T_{i}a-T_{i}b\right]}^{2}}, \end{array}$$

(5) $$\begin{array}{c} D_{T}=\mathrm{Max}\left(\text{Hit}\right)+XS_{\text{Dev}}. \end{array}$$

A selectivity parameter (*S*) is used to determine whether the qualitative model is applicative or not. *S* is calculated as formula (6). When *S* > 1, the classes of 1 and 2 can be completely separated; when *S* = 1, the classes can be separated with warning. The larger the *S* value is, the greater the discrimination result and the accuracy of the prediction model will be [17]:

(6) $$\begin{array}{c} S=\frac{D}{\left(D_{T1}+D_{T2}\right)}. \end{array}$$

## 3. Results and Discussion

### 3.1. Discrimination of Fritillaries

#### 3.1.1. Selection of Spectral Pretreatment Method

As shown in Figure 3, the original NIR spectra for different species of Fritillaria are highly similar. The spectrum band between 4000 and 9000 cm^−1^ is often intercepted for analysis due to its abundant information and high intensity. However, there is severe band overlap, making it difficult to interpret spectral information. Therefore, spectral pretreatment is a critical step in expanding the differences between the spectra of each sample, extracting more effective spectral information, and improving the signal-to-noise ratio.

In this paper, the first derivative method was used for spectral pretreatment, and the scaling to first range method and factorization method were used as the algorithm, respectively. The smoothing point was set at 13, and spectrum band between 4000 and 5000 cm^−1^ was selected for analysis.

#### 3.1.2. Establishment of Qualitative Analysis Model

When the factorization method was used as the algorithm, the analysis result showed that *S* is below 2, indicating that the accuracy of qualitative classification for unknown samples is too low. While the first derivative method was used, the qualitative analysis results of the model were satisfactory (Table 1). Table 1 shows the smallest *S* values among all species of Fritillaria, and each *S* value exceeds 2, which suggests that all species of Fritillaria can be completely distinguished. Fourteen Fritillaria samples were used to test the qualitative NIR model. As a result, the mistaken judgment rate of the qualitative NIR model is zero.

### 3.2. Quantitative Analysis of the Total Alkaloids from Fritillaria

#### 3.2.1. Content Determination of the Total Alkaloids by UV Spectroscopy

Acid dye colorimetry has been a commonly used and well-recognized method for the alkaloid content of plants. Its analysis results are used as the true values for quantitative analysis of Fritillaria in this study. Fritillaria alkaloids can form yellow ion pairs with bromothymol blue at pH 5 buffer solutions, and the ion pairs are quantitatively extracted by chloroform. Peiminine is the main component of Fritillaria alkaloids and was therefore selected as the reference compound with the maximum UV absorption of ion pairs in chloroform at 410 nm. The linear regression equation was obtained (*A* = 0.000541 + 0.040201*C*, *r* = 0.9998) with good linearity within the concentration range of 1.78~8.90 *μ*g/mL.

The quantitative analysis results of seven samples were shown in Table 2.

#### 3.2.2. Establishment of NIR Quantitative Model

Based on the pretreated NIR spectra, the NIR quantitative analysis model for the total alkaloids from Fritillaria was established using the PLS method with UV analysis data as the true values.

OPUS software provides two methods for validation of the quantitative model: internal cross validation and external validation. Due to the limited samples used in this experiment, the internal cross validation was used to optimize the model. We used RMSECV values as assessment criteria to acquire the optimal frequency range and PLS components.

The influences of five different PLS spectral pretreatment methods on the NIR quantitative model were listed in Table 3. When Min-Max normalization was adopted, the spectra were shifted linearly, so that the minimum *Y*-value equals zero. Meanwhile the spectra were expanded, so that the maximum *Y*-value equals two absorbance units. This method is comparable to vector normalization. Multiplicative scatter correction is often used for measurements in diffuse reflection, while vector normalization is used to eliminate the influence of different optical path lengths in case of transmission measurements. Subtraction of a straight line is used to eliminate linear baseline shifts, which result from different values of the detector amplification. By calculating the first derivative, a relative enhancement of the sharp structures compared to the original spectrum can be observed and the signals with steep edges get more emphasis than relatively flat bands. Thus the first derivative method can be used to emphasize pronounced but small features compared to huge broad-banded structures and leads to the best calibration results.

The results in Table 1 show that the correlation coefficient (*R* ^2^) and RMSECV values by using five different spectral pretreatment methods have little distinction in the optimal frequency range of 6082.8–4165.8 cm^−1^. In this study, the first derivative was selected as the spectral pretreatment method for the quantitative model.

**Figure 4:**
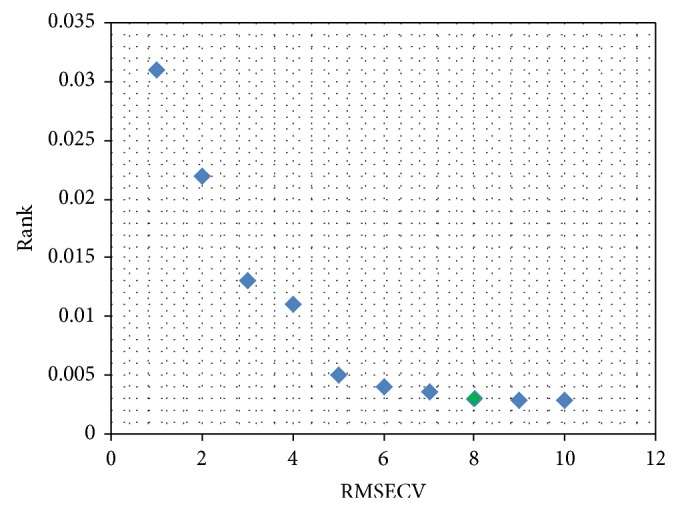
Determination of optimal PLS components by cross validation.

Optimization of the PLS components was shown in Figure 4. The final results of the model optimization are as follows: PLS spectral pretreatment methods: first derivative method; optimal frequency range: 4165.8~6082.8 cm^−1^; optimal PLS components: 8.

#### 3.2.3. Quantitative Prediction by PLS Model

The established PLS model was used to predict the total alkaloid contents of 28 samples in validation set, and the corresponding prediction values were obtained (Table 4). The values of RMSEP, *R* ^2^, and SEP for the prediction results were 0.00643, 0.9893, and 0.00702, respectively. To validate the precision of the established NIRS models, SEP should be assessed in relation to the precision of the reference method illustrated as the standard error of the laboratory (SEL), and the calculating formulae can be found in [17]. In this work, SEL was calculated to be 0.0051, and SEP was 0.00702 which was less than 2 × SEL, suggesting the precision of the established NIR model was satisfactory for total Fritillaria alkaloid contents analysis. The largest deviation between NIR prediction value and UV true value is 3.3%, which indicates that the established PLS model can give accurate prediction.

The NIR method and the UV method can be compared with a paired *t*-test. In the 95% confidence interval, the value of statistics *t*_(0.05)_ = 0.016 was less than the corresponding critical value *t*_(0.05,27)_ = 2.052. The results demonstrated that there was no significant difference between the NIR and the UV method.

#### 3.2.4. Results of Quantitative Analysis by PLS Model

In order to validate the accuracy and applicability of the PLS model, seven kinds of Fritillaria powder samples in the verification set were scanned to acquire NIR spectra. The quantitative prediction values for seven samples were obtained by using established PLS models (Table 5). In the 95% confidence interval, the value of statistics *t*_(0.05)_ = 0.020, which was less than the corresponding critical value, *t*_(0.05,6)_ = 2.447, suggests that there was no significant difference between the NIR and the UV method.

Compared with UV method in quantitative analysis for total alkaloid content of Fritillaria, NIRS method is simpler, faster, and more efficient, due to its nondestructive character. Unlike in the analysis with UV method, the tedious sample treatments were completely avoided in NIR analysis.

## 4. Conclusion

In this study, a qualitative NIR model for discrimination of seven kinds of Fritillaria was established with the first derivative as the spectra pretreatment method. The scaling to first range worked as the algorithm and 4000 to 5000 cm^−1^ as the scanning wavelength range with 13 points smoothing. The qualitative NIR model was verified with 14 Fritillaria samples. As a result, the mistaken judgment rate of the qualitative NIR model is zero.

A quantitative NIR model for total alkaloids from Fritillaria was established by using the PLS method with UV analysis data as the true values. The first derivative method was used for the spectra pretreatment and the internal cross validation was used to optimize the model. RMSECV values served as assessment criteria to acquire the optimal frequency range and PLS components. The established quantitative NIR model can accurately predict the content of total alkaloids from Fritillaria samples with a correlation coefficient of 0.9935 and a RMSECV of 0.00339. The paired *t*-test between NIR prediction values and UV values indicated that there was no significant difference between NIR and UV methods.

As the experiment has shown, the NIR analysis methods developed here provide a new, simple, fast, and accurate approach for the qualitative and quantitative analysis of Fritillaria.

## Figures and Tables

**Table 1:** The qualitative analysis results of the model using the first derivative method.

Number	Group 1	Group 2^a^	*S*
1	*F. thunbergii* Miq. (big)	*F. pallidiflora* Schrenk	2.208536
2	*F. thunbergii *Miq. (small)	*F. pallidiflora* Schrenk	10.515240
3	*F. ussuriensis* Maxim	*F. pallidiflora* Schrenk	3.510903
4	*F. delavayi* Franch	*F. cirrhosa* D. Don	12.225511
5	*F. unibraacteate* Hsiao et. K. C. Hsia	*F. pallidiflora* Schrenk	8.895630
6	*F. cirrhosa* D. Don	*F. delavayi* Franch	12.225511
7	*F. pallidiflora* Schrenk	*F. thunbergii* Miq (big)	2.208536

^a^Fritillaria types in Group 2 are the most similar varieties to Fritillaria in Group 1, respectively. *F. thunbergii* Miq. (big) and *F. pallidiflora* Schrenk are the most similar species to each other, so the *S* values in number 1 and number 7 are the same (2.208536). *F. delavayi* Franch and *F. cirrhosa* D. Don are also in the same case.

**Table 2:** Content of total alkaloids from seven Fritillaria samples by UV spectroscopy.

Number	Sample	Absorbance	Total alkaloids (%)
1	*Fritillaria ussuriensis* Maxim	0.282	0.1750
2	*Fritillaria thunbergii* Miq. (big)	0.387	0.2406
3	*Fritillaria thunbergii *Miq. (small)	0.344	0.2138
4	*Fritillaria pallidiflora* Schrenk	0.392	0.2430
5	*Fritillaria cirrhosa* D. Don	0.187	0.1160
6	*Fritillaria unibraacteate* Hsiao et. K. C. Hsia	0.327	0.2030
7	*Fritillaria delavayi* Franch	0.263	0.1640

**Table 3:** Model verification results by using different spectral pretreatment methods.

Number	Spectral pretreatment methods	PLS components	*R* ^2^	RMSECV
1	Min-Max normalization method	7	0.9944	0.00314
2	Multiplicative scatter correction method	6	0.9943	0.00319
3	Vector normalization method	6	0.9942	0.00320
4	First derivative method	8	0.9935	0.00339
5	Subtraction of a straight line	7	0.9939	0.00378

**Table 4:** Quantitative predictions for the samples in prediction set by PLS model.

Number	Sample	NIR prediction value/%	UV true value/%	Deviation
1	*Fritillaria ussuriensis* Maxim	0.1762	0.1750	−0.001240
2	*Fritillaria ussuriensis* Maxim	0.1757	0.1750	−0.000653
3	*Fritillaria ussuriensis* Maxim	0.1722	0.1750	0.002770
4	*Fritillaria ussuriensis* Maxim	0.1761	0.1750	−0.001110
5	*Fritillaria thunbergii* Miq. (big)	0.2386	0.2406	0.002030
6	*Fritillaria thunbergii* Miq. (big)	0.2454	0.2406	−0.004770
7	*Fritillaria thunbergii* Miq. (big)	0.2355	0.2406	0.005060
8	*Fritillaria thunbergii* Miq. (big)	0.2427	0.2406	−0.002070
9	*Fritillaria thunbergii *Miq. (small)	0.2110	0.2138	0.002800
10	*Fritillaria thunbergii *Miq. (small)	0.2170	0.2138	−0.003230
11	*Fritillaria thunbergii *Miq. (small)	0.2160	0.2138	0.002270
12	*Fritillaria thunbergii *Miq. (small)	0.2099	0.2138	0.003890
13	*Fritillaria pallidiflora* Schrenk	0.2416	0.2430	0.001350
14	*Fritillaria pallidiflora* Schrenk	0.2383	0.2430	0.004680
15	*Fritillaria pallidiflora* Schrenk	0.2416	0.2430	0.001350
16	*Fritillaria pallidiflora* Schrenk	0.2513	0.2430	−0.008280
17	*Fritillaria cirrhosa* D. Don	0.1167	0.1160	−0.0006750
18	*Fritillaria cirrhosa* D. Don	0.1148	0.1160	0.001240
19	*Fritillaria cirrhosa* D. Don	0.1154	0.1160	0.000615
20	*Fritillaria cirrhosa* D. Don	0.1188	0.1160	−0.002750
21	*Fritillaria unibraacteate* Hsiao	0.1967	0.2030	0.006280
22	*Fritillaria unibraacteate* Hsiao	0.2079	0.2030	−0.004920
23	*Fritillaria unibraacteate* Hsiao	0.1978	0.2030	0.005160
24	*Fritillaria unibraacteate* Hsiao	0.2071	0.2030	−0.004100
25	*Fritillaria delavayi* Franch	0.1657	0.1640	−0.001720
26	*Fritillaria delavayi* Franch	0.1632	0.1640	0.000758
27	*Fritillaria delavayi* Franch	0.1637	0.1640	0.000307
28	*Fritillaria delavayi* Franch	0.1650	0.1640	−0.001040

**Table 5:** Quantitative analysis results for the samples in validation set by PLS model.

Number	Sample	NIR prediction value/%	UV true value/%
1	*Fritillaria ussuriensis* Maxim	0.1728	0.1640
2	*Fritillaria thunbergii* Miq. (big)	0.2375	0.2030
3	*Fritillaria thunbergii *Miq. (small)	0.2165	0.1160
4	*Fritillaria pallidiflora* Schrenk	0.2411	0.2430
5	*Fritillaria cirrhosa* D. Don	0.1165	0.1750
6	*Fritillaria unibraacteate* Hsiao	0.2080	0.2138
7	*Fritillaria delavayi* Franch	0.1661	0.2406
